# Supplementary material for: Non-1st seizure was less severe than 1st seizure with non-urgent level among suspected seizures transferred by ambulance
Source: PLoS One. 2023 Aug 29;18(8):e0290783. doi: 10.1371/journal.pone.0290783 (PMC10464987; doi:10.1371/journal.pone.0290783)
Supplement: Attachment — Submitted filename: ResponseToReviewers_R1.docx [file pone.0290783.s001.docx]

Response to the comments from the reviewers

We greatly appreciate the thorough review and kind advice, which have helped us to improve the quality of our manuscript. We are sorry for the delay in providing our responses to the questions raised, as we needed time to revise the text. The manuscript has been amended to clarify the issues raised by the reviewer. Our changes and point-by-point responses to the comments made are summarized below. We have also tracked the changes in red in the revised manuscript.

Reviewer #1:

Authors compare patients with suspected seizures on their first transfer by ambulance and those who had been transferred ≥ 2 times. During a 5 y retrospective study In this study 636 patients were evaluated and authors conclude that seizure severity was lower in the Non-1 st Group than in the 1 st seizure Group .The subject is of interest, I have following comments
1. Abstract: Should not have introduction, instead a line or two may be given as background and state the aim of the study .

Response:

We have amended the Abstract as suggested.

2. The conclusion in abstract is too long , should state in a line or tow

Response:

We have rephrased the suggested parts.

Introduction: “In Status epilepticus neuronal injury Is the main concern” in my opinion physical injury is also very important probably more important than neuronal injury

Response:

We have clarified this issue in the Introduction section. We have also added a phrase citing tree references in the Discussion section.

Result: The result is repetitive and repeats the findings in Table 1, 2 and 3 .I suggest the main findings should be briefly stated and referred to the respective table, .Similarly the statistical tests used in the analysis should not be re stated as they have been mentioned in statistical method section or the tests may be mentioned in respective Table as footnote

Response:

We are sorry for the redundant descriptions in the Results section. We have added subsection 3.1 Main findings in the Results section.

The discussion is not focussed, I would suggest that briefly summarise the major findings in a line or tow and discus the results in the light of published information /literature
It is a good study, well conducted but I would like it to be shorter to make it more readable and less repetitive.

Response:

We agree with these comments and have added key results.

Reviewer #2:

Asano et al. present a manuscript aiming to compare the severity of seizures between patients who are transferred to the hospital by ambulance for the first seizure and patients who are transferred for a second or later seizure. The reason for asking this question was to inform guidelines about the requirement for an ambulance call after seizures depending on if it was a first or a later seizure. The authors report that first-time seizures were more severe and argue that this supports current guidelines which do not necessarily require calling an ambulance for the second or later seizures.

The data is principally interesting but seizure severity may not be the main factor that should drive the decision if an ambulance need to be called. Even if a first seizure was not severe, the fact that there may be an acute cause for the first event requiring urgent treatment suggests that first seizures should be worked up urgently. On the other hand, if the diagnosis of epilepsy is known and the seizures are typical, then there is very unlikely an acute symptomatic cause, i.e. the decision to transport to the hospital depends on event severity. Therefore, assessing severity of the second or later seizure as done in this study is reasonable but I do not see why the comparison to the severity of the first event is helpful as these need to be seen urgently anyway even if some patients do not need to be admitted to the hospital once an acute cause has been excluded. Of note, 5% of the patients presenting after the first seizure had status epilepticus or required admission to the ICU which was similar to the patients after the second or later seizure (4.8%). There was a lower percentage which required admission to the hospital overall after the second seizure, probably as the general epilepsy workup had already been completed in the past. This may not reflect "severity" as interpreted in the study.

Response:

Since ED physicians do not perform the general workup for epilepsy, patients who were transferred to ED by ambulance are referred to general neurological physicians. Then, if those clinicians consider that the patient needs to see an epilepsy specialist, they are referred to an epilepsy specialist in our facility. We have added this information to the revised text.

There are also methodological issues that need to be addressed.
-The inclusion criteria are not clear to me. The authors list 4 different inclusion criteria that are combined with "or". Criterion 1 is "patients transferred due to possible CNS disorders". This would mean everybody with a neurological cause would be included which does not make sense.

Response:

We agree that our initial descriptions were unclear and have rephrased the part in question.

They later list "keywords" (page 15/16) which are not keywords but rather search criteria. But again, these do not help much to understand the selection process. Did they include patients transported to the ED AND seen by epilepsy specialists? Then they would have missed the ones that were not seen by epilepsy specialists. The authors need to very clearly state how they selected the included patients and which search criteria they used.

Response:

We have changed the “keywords” to “search criteria”. For the explanation of “patients transported to the ED AND seen by epilepsy specialists” were included in criterion: 1) who were considered in need of attention from an epilepsy specialist among ED physicians. We have added some explanations about our system.

- The authors use an ordinal scale to describe "severity". They also comment that they used the U test as the data did not follow normal distribution. However, they calculate mean and standard deviation for this ordinal scale which does not make sense.

Response:

We have changed the data provided from mean and SD to median and interquartile range.

- It is confusing that a numbered scale is also used for the transport reasons. This scale is clearly not ordinal so it may be better to remove the numbers. Also, it appears to me that this "reason for transport" was not the reason used at the time the decision for transport was made but rather later identified. For example, PNES was defined by ED physicians (page 15) so could not have been a reason for transport. I wonder if the authors rather want to describe the cause for the transport?

Response:

We have removed the numbers in accordance with these comments.

We have also changed the phrase “reason for transport” to “etiology after transport”.

Other issues:
- The authors should provide information how many of their patients overlap between the group with first seizures and the group with 2nd and later seizure.

Response:

This represents an important point, but might be a different topic regarding the hypothesis in this study. We have been collecting data about unprovoked seizures and epilepsy, with the intention of writing another paper. We hope the reviewer will understand.

- The severity score is used in the abstract but the reader does not know the definition.

Response:

We have added the definition in the Abstract.

Reviewer #3:

This paper was aimed to compare severity of suspected seizures between two groups of patients, i.e those with suspected seizures transferred by ambulance for either the first time (1st Group) or at least the second time (Non-1st Group). The grade of suspected seizure severity was lower in the Non-1st Group than in the 1st Group. These findings confirm previous literature reports that ambulance transport is often unnecessary, and suggest that other strategies might be useful for patients with suspected seizures.

Epidemiological data are described in detail, and well addressed in the Discussion. Statistical analysis is adequate.

Response:

Thank you very much for reviewing our manuscript. We appreciate your time and effort in providing advice on our paper.

Reviewer #4:

The current data and results do not fully support the conclusion.
1) Table 4 shows significant difference in severity grade 3 and grade 4 in 1st and Non-1st Groups. How about the severity grade for the Non-1St Group at the first ambulance transport only? Is there any difference compared to those of 1st group and those of Not-1st groups at the following transports.

Response:

This is an interesting point. Since the primary endpoint was a comparison between 1^st^ and Non-1^st^ Groups, comparisons of severity between the 1^st^ Group and first transport of the Non-1^st^ Group might be better discussed in another study. As described in Table 5, ASS was higher and PNES was less in the 1^st^ Group than in the Non-1^st^ Group. We hope this response is acceptable.

2) In Conclusion, authors note that "About 95% of Non-1st Group suspected seizures were more than grade 4". Most patients who are in Non-1st group are on Anti-seizure medication (ASM). Patients may be instructed to follow up with their neurologists once their conditions are stable at ER. Will this affect severity assessment in this study?

Response:

Thank you very much for raising this important suggestion. Since etiology after trip (Table 5) showed a frequency of definitive seizures of 67% in the 1^st^ Group and 65% in Non-1^st^ Group, we thought that taking ASM might not have affected severity.

3) It is grateful if authors can present more details on how to reduce unnecessary use of ambulances on page 23.

Response:

This is another important suggestion. We plan to provide a card as a simplified version of Table 6 that could provide a graphic summary that people can easily understand and ask patients with epilepsy to always carry this card with them. We have added this explanation to the Discussion section.

Reviewer #5:

The study is interesting and well-written. However, in my opinion there are major shortcomings to be addressed in order to be considered for publication:
INTRODUCTION
• “The reason for timing seizures is that seizures lasting >5 min can be considered status epilepticus (SE)” Please specify whether you are speaking of convulsive seizures. Otherwise, change your timeline (see “A definition and classification of status epilepticus – Report of the ILAE Task Force on Classification of Status Epilepticus” doi: 10.1111/epi.13121).

Response:

We meant convulsive seizures and have added “convulsive” before “seizures”.

• However, the problem of inappropriate or unnecessary ambulance calls is a problem in the real world. Please double check repetitions (e.g. problem in this sentence)

Response:

Thank you for pointing this out. We have modified the text to read: “inappropriate or unnecessary ambulance calls are a problem in the real world.”

METHODS
• I do not understand whether you explored only tonic-clonic seizures and mimics or included all seizure types. Should you focus on convulsive semiology, you should opt between these two options (1) convulsive seizures and all mimics (e.g. syncope, not only PNES (2) convulsive seizures only – it is a retrospective study, you can choose.

Response:

We have added the sentence, “We included patients not only with tonic-clonic seizures but also patients with other seizure types or mimics (psychogenic non-epileptic seizure [PNES] and syncope)”.

• “in need of attention from an epilepsy specialist”. How could you evaluate this criterium? As a referral after general neurological consultancy at the ED? If so, please specify how referrals are managed at your site and whether you can actually distinguish between referrals to neurologists (generally speaking) and epileptologists. Please clarify how the neurologcy emergency service is organized at your Hospital.

Response:

We have added our system for referrals.

• “patients with psychiatric symptoms including psychogenic non-epileptic seizure (PNES)”. The diagnosis of PNES should be referred to published guidelines (see “Classification and comparative analysis of psychogenic nonepileptic seizures (PNES) semiology based on video-electroencephalography (VEEG)” doi: 10.1016/j.yebeh.2020.107697)? In my opinion it is redundant herein, this point can be included within number 1 inclusion criterium

Response:

We have added the sentence, “The patients with PNES were diagnosed with long-term video-electroencephalography” along with the suggested reference. Since including the first inclusion criterion was difficult, we have separated this content on PNES from the statement about inclusion criteria. We hope the text is now easier to understand.

• Exclusion criteria: “3) patients who had apparent nonepileptic, CNS disorders such as subarachnoid or intracranial hemorrhage, chronic subdural hemorrhage, CNS infection, etc.;” This collides with the first inclusion criterium. For instance, acute symptomatic seizures can result from encephalitis and non-convulsive seizures may be hardly distinguishable from other acute CNS disordersf

Response:

Thank you for pointing out this overlap. As this sentence may have caused some confusion, we have rephrased the text and trust that the modified sentence is more understandable.

• “We conducted comparisons using the most severe grade for the Non-1st Group, rather than mean or median in uni- and multivariate logistic regression analysis”. Please explicate the meaning of “Most severe grade”. If not better justified, mean values should be included in the analysis to draw firm conclusions.

Response:

Thank you for pointing this out. In the Non-1^st^ Group, some patients were transferred many times during the study period. For these patients, we investigated grade of severity for each ambulance visit and used the highest grade of severity for the purposes of comparison. We have modified the sentence as follows: “If Non-1^st^ Group patients were transferred twice or more, we conducted comparisons using the most severe grade rather than mean or median in uni- and multivariate logistic regression analysis.”

RESULTS
• Please clarify you did not count multiple times patients in the non 1st seizure group

Response:

Thank you for this pertinent suggestion. We have added the explanation in 2.4.1.

• Is there any difference for paediatric patients?

Response:

We did not investigate differences between the settings of pediatric and adult patients. We are therefore unable to answer this question.

DISCUSSION
• “In this study, the fact that most trips by the Non-1st Group were for grades 4 and 5, and only 0.2% of trips required resuscitation and 3% required admission to the intensive care unit is also worth knowing. Since we did not study whether specific trips were unnecessary in this study, we cannot conclude that most trips for the Non-1st Group were unnecessary. However, the collected data revealed that most trips for the Non-1st Group did not require admission to hospital”. Where do these data come from? There is no mention in the results

Response:

In both grades 4 and 5, patients did not need hospitalization. These data come from the fact that more than 95% of the Non-1^st^ Group was classified as grade 4 or 5. We have modified the sentence as follows, “In this study, the fact that most trips by the Non-1st Group were for grades 4 and 5, which do not require admission to the hospital, and only 0.2% of trips required resuscitation.”

• A discussion on possible seizure precipitants in patients with established epilepsy should be considered, as possible trigger of seizures should be taken into account when assessing the non 1st seizure group (see “ Dealing with the storm: An overview of seizure precipitants and spontaneous seizure worsening in drug-resistant epilepsy” doi: 10.1016/j.yebeh.2019.05.036)

Response:

We have added these sentences to the Limitations: “This study did not investigate the effect of treatment changes or provocative factors such as stress, sleep deprivation, and alcohol on seizure occurrence, although these factors are reported to aggravate seizures. Studies that include these factors may be desired.”

TABLES AND FIGURES
Please add figures that may help the readership in interpreting the results (especially for the logit analysis).

Response:

If accepted for publication, we will consider making a graphic summary figure.
