# Supplementary material for: Non-1st seizure was less severe than 1st seizure with non-urgent level among suspected seizures transferred by ambulance
Source: PLoS One. 2023 Aug 29;18(8):e0290783. doi: 10.1371/journal.pone.0290783 (PMC10464987; doi:10.1371/journal.pone.0290783)
Supplement: Attachment — Submitted filename: ResponseToReviewers_R2.docx [file pone.0290783.s002.docx]

Response to the comments from the reviewers

We would like to extend our sincere thanks for your efforts in the second round of reviews. Our gratitude to all the reviewers for their thorough and detailed evaluations cannot be overstated. We are pleased to note that Reviewers #3, #4, and #5 have given their approval to the current manuscript. However, we wish to inform you that we are contemplating further revisions to incorporate the constructive feedback from Reviewer #2. We are aware that these modifications will introduce changes to the current content, yet we trust that the insights provided by Reviewer #2 are crucial for our research. We humbly seek your understanding in this regard.

As per the feedback of Reviewer #2, we have adjusted our responses accordingly, which we detail below. We would also like to take this opportunity to express our deep appreciation for the invaluable time and intellectual contribution of all the reviewers. It is indeed the insightful feedback we have received that has refined and honed our paper scientifically, for which we convey our deepest gratitude.

I had two main concerns that were not addressed by the authors:
- "seizure severity may not be the main factor that should drive the decision if an ambulance need to be called. Even if a first seizure was not severe, the fact that there may be an acute cause for the first event requiring urgent treatment suggests that first seizures should be worked up urgently": This could be mentioned in the introduction and then a statement made that this does not apply anymore for the second seizure and that the authors are, therefore, looking at severity of the actual event.

Response:

Thank you for your feedback. We apologize for not fully understanding the two points you highlighted. In accordance with your advice, We have added the aforementioned text to the Introduction.

"capturing the 4.8% patients even after a 2nd seizure who need ICU admission may be very important and therefore the transport justified!": This must be pointed out in the discussion. The authors should comment if this can be recognized by the ambulance i.e. if these all had severity 1-2. If yes, a recommendation could be made which severity score would trigger transport to the hospital with a 2nd seizure. In the current form, the manuscript gives no recommendation regarding this and the reader may (possibly appropriately) think that a transfer after a 2nd seizure is needed to capture the 4.8% who are severely ill.
Response:

We have modified a part of the Discussion and incorporated your pointed suggestions.

The authors answered to the above two questions that patients are worked up in ED by general neurology and only if needed an epilepsy specialist is called. This is the response to a different concern I had but the two main concerns I mentioned above are not addressed.

Response:

We deeply apologize for failing to respond to your two questions. As you pointed out in your comments, we have made corrections in agreement with your feedback

Additional issues arising from the answers to my or the other reviewer's questions:
- A major issue is that they only looked at patients who were eventually referred to an epileptologist. Patients taken care of by neurology and not referred to an epileptologist were not included. So, if a patient was found in ED to have an intracranial bleeding causing the seizure, they could have been treated without an epileptologist being involved as the pathway was clear and there was no need to involve an epileptologist. Missing such a severe etiology would certainly bias the study. In addition, the ED physician or neurologist may not have felt the need to call the epileptologist in case of an established epilepsy. Furthermore, they excluded "patients who had already been diagnosed with an established epilepsy at other facilities and first transfer to our facility". The latter would certainly fit the criteria for patients with a second or later seizure. This means that the cohort in this study is biased and may not be appropriate to answer the question.

Response:

Thank you for your comments. In this study, we are looking at the difference in severity between 1^st^ Group and Non-1^st^ Group among patients who presented with suspected seizures. As you pointed out, even if there are seizures, severe conditions like stroke which are apparent in neuro-imaging usually have significantly different treatment paths and would typically be handed over from the ER to neurology or neurosurgery. Therefore, we decided to exclude them, assuming that they wouldn’t usually be treated as suspected seizures. We appreciate your understanding.

- The inclusion and exclusion criteria are very confusing and do not clearly state the cohort that was included in the study. For example. the exclusion criteria include "patients who had apparent non-epileptic CNS disorders such as subarachnoid or intracranial hemorrhage, chronic subdural hemorrhage, CNS infection". This is confusing. Do they mean patients who did not have seizures but these disorders? Of course these should not be included if they did not have seizures but this should then rather be handled by them not fulfilling the INCLUSION criteria. As this is a study looking at seizures, the inclusion criteria should state "patients with seizures who were 1) considered in need of attention from an epilepsy specialist among ED physicians..." etc. It is fine if they screened more patients but of course if they did not have seizures they were not included.

Response:

Your point is indeed very understandable. In this study, we excluded cases of seizure-onset stroke in our criteria. However, even if the stroke onset was initiated by a seizure, if the emergency examinations in the ER indicate that it is an acute symptomatic seizure caused by a stroke or an infection, we believe it would not be considered a ‘suspected seizure’, and there would rarely be a need for consultation with an epilepsy specialist. This may overlap with the previous answer, but we judged these exclusion criteria to be reasonable for this study.

- "All participants provided written or verbal informed consent prior to inclusion in the study". If this is correct, the authors must have excluded any patient who did not recover or passed away as they would not have been able to provide consent. This would have introduced additional bias.
Response:

This is an important observation, and without your suggestion, it would have gone unnoticed. We appreciate your input. With regards to this point, we have added an appropriate comment to the Method section.

In conclusion, I am quite concerned that the cohort included in the study may not be representative of the cohort the authors would like to make recommendations for.

Response:

We might be repeating ourselves here, but this study is specifically focused on cases where epileptic seizures are suspected, not conditions presenting with acute symptomatic seizures caused by severe condition. I hope you understand that the scope of this research is not as broad as what Reviewer #2 might be concerned about. I believe from the title of this paper; readers will understand that the study is limited to suspected seizures.

On the whole, we deeply appreciate your valuable comments, which have significantly improved our manuscript.

Reviewer #3: The Authors addressed almost ll the queries and comments of the reviewers, and I think that this paper is now improved.

Response:

Thank you for spending your valuable time on this. Thanks to your input, we were able to make many improvements. We deeply appreciate it.

Reviewer #4: All comments are addressed and acceptable. The current manuscript meets the requirement for publication.

Response:

Thank you for spending your valuable time on this. Thanks to your input, we were able to make many improvements. We deeply appreciate it.

Reviewer #5: Dear Authors,
you have successfully addressed the uncertainties of the first draft.
I suggest you to draw a figure summarizing the Results of the logit analysis.

Response:

Thank you for spending your valuable time on this. Thanks to your input, we were able to make many improvements. We deeply appreciate it. We currently have six tables in our manuscript, and as authors, we would prefer not to add more figures to avoid complexity. We hope for your understanding in this matter. However, if you believe a figure is necessary, we are more than willing to create one. In that case, we kindly ask for your advice.
